# Supplementary material for: ZIKA Virus infection in pregnant women in French Guiana: More precarious-more at risk
Source: PLoS Negl Trop Dis. 2020 Mar 24;14(3):e0008193. doi: 10.1371/journal.pntd.0008193 (PMC7122809; doi:10.1371/journal.pntd.0008193)
Supplement: S1 Checklist — (DOC) [file pntd.0008193.s001.doc]

STROBE Statement—checklist of items that should be included in reports of observational studies

|  | Item No | Recommendation |
| --- | --- | --- |
| **Title and abstract** | 1 | (*a*) Indicate the study’s design with a commonly used term in the title or the abstract |
|  |  | (*b*) Provide in the abstract an informative and balanced summary of what was done and what was found  “A monocentric retrospective case-control study…” |
| Introduction | | |
| Background/rationale | 2 | Explain the scientific background and rationale for the investigation being reported  “Poor neighborhoods, with informal housing, lack of sanitation and presence of vector breeding places are especially suited for *Aedes aegypti*, the main vector for arboviruses in FG. This urban mosquito preferentially breeds around human dwellings, in outdoor water storage containers and in any recipient containing stagnant rain water. Densely populated areas with sustained human activity during the day are perfect for a daytime feeder that can bite several people in a short period of time” |
| Objectives | 3 | State specific objectives, including any prespecified hypotheses  “allowed us to test whether the poorest pregnant women were more likely to acquire ZIKV than more socioeconomically privileged women.” |
| Methods | | |
| Study design | 4 | Present key elements of study design early in the paper  “A multicentric, cohort was conducted in Cayenne (CHAR), Kourou (CHK) and Saint Laurent (CHOG) hospital, the main cities and the main hospitals in FG.  ” |
| Setting | 5 | Describe the setting, locations, and relevant dates, including periods of recruitment, exposure, follow-up, and data collection  “**Population.** Were included **a**ll deliveries or medical abortions between January 2016 and December 2016, 3 months after the official declaration of the end of the outbreak. Women without ZIKV serology were excluded. We also included women with positive RT-PCR even if they did not have any serology. ” |
| Participants | 6 | 1. *Cohort study*—Give the eligibility criteria, and the sources and methods of selection of participants. Describe methods of follow-up   *“See above”*  *Case-control study*—Give the eligibility criteria, and the sources and methods of case ascertainment and control selection. Give the rationale for the choice of cases and controls  *Cross-sectional study*—Give the eligibility criteria, and the sources and methods of selection of participants |
|  |  | (*b*) *Cohort study*—For matched studies, give matching criteria and number of exposed and unexposed  *Case-control study*—For matched studies, give matching criteria and the number of controls per case |
| Variables | 7 | Clearly define all outcomes, exposures, predictors, potential confounders, and effect modifiers. Give diagnostic criteria, if applicable  “The following variables were available: age, area of residence, pregnancy trimester and pregnancy outcomes (type, date, attendant, place, and last menstruation date), and health assurance.” |
| Data sources/ measurement | 8* | For each variable of interest, give sources of data and details of methods of assessment (measurement). Describe comparability of assessment methods if there is more than one group  “For health insurance status, we distinguished between patients in a precarious social situation and those who were not. Patients without any health insurance [9] , those benefiting from free universal health care called “CMU” (which allows access to health for person who have resided legally in France for more than 3 months and who are not already covered), or those benefiting from “State medical aid” or “AME” (government run insurance program specifically conceived for undocumented migrants who become eligible after 3 months of residency in a French territory) were considered to be in a precarious social situation. Persons with regular social security were considered non precarious.” Serum samples (serum and urine for RT-PCR),obtained during trimestrial surveillance and during possible acute symptomatic illnesses or in presence of structural abnormalities or fetal death, placenta and amniotic liquid were assayed for ZIKV RNA by real time RT-PCR using the Lanciotti method [10] or RealStar® ZIKV RT-PCR kit or ELISA serology. Analyses were realized by the NRC of Pasteur Institute in French Guiana and by the laboratory of Cayenne Hospital Center. Neutralisation for IgG could not be implemented, therefore we excluded probable cases with negative IgM and positive igG. |
| Bias | 9 | Describe any efforts to address potential sources of bias |
| Study size | 10 | Explain how the study size was arrived at  “Were included **a**ll deliveries or medical abortions between January 2016 and December 2016, 3 months after the official declaration of the end of the outbreak” |
| Quantitative variables | 11 | Explain how quantitative variables were handled in the analyses. If applicable, describe which groupings were chosen and why |
| Statistical methods | 12 | (*a*) Describe all statistical methods, including those used to control for confounding |
|  |  | (*b*) Describe any methods used to examine subgroups and interactions |
|  |  | (*c*) Explain how missing data were addressed |
|  |  | (*d*) *Cohort study*—If applicable, explain how loss to follow-up was addressed  *Case-control study*—If applicable, explain how matching of cases and controls was addressed  *Cross-sectional study*—If applicable, describe analytical methods taking account of sampling strategy |
|  |  | (*e*) Describe any sensitivity analyses |

Continued on next page

Bivariate analysis of categorical variables used Poisson regression to obtain prevalence ratios rather than odds ratios, which may overestimate the magnitude of association . Available variables were used in the multivariate analysis using modified Poisson regression in order to obtain prevalence ratios.

| Results | | |
| --- | --- | --- |
| Participants | 13* | (a) Report numbers of individuals at each stage of study—eg numbers potentially eligible, examined for eligibility, confirmed eligible, included in the study, completing follow-up, and analysed “fig 1 and table 1) |
|  |  | (b) Give reasons for non-participation at each stage |
|  |  | (c) Consider use of a flow diagram |
| Descriptive data | 14* | (a) Give characteristics of study participants (eg demographic, clinical, social) and information on exposures and potential confounders |
|  |  | (b) Indicate number of participants with missing data for each variable of interest |
|  |  | (c) *Cohort study*—Summarise follow-up time (eg, average and total amount) |
| Outcome data | 15* | *Cohort study*—Report numbers of outcome events or summary measures over time  Table 1 |
|  |  | *Case-control study—*Report numbers in each exposure category, or summary measures of exposure |
|  |  | *Cross-sectional study—*Report numbers of outcome events or summary measures |
| Main results | 16 | 1. Give unadjusted estimates and, if applicable, confounder-adjusted estimates and their precision (eg, 95% confidence interval). Make clear which confounders were adjusted for and why they were included 2. Table 1 |
|  |  | (*b*) Report category boundaries when continuous variables were categorized |
|  |  | (*c*) If relevant, consider translating estimates of relative risk into absolute risk for a meaningful time period |
| Other analyses | 17 | Report other analyses done—eg analyses of subgroups and interactions, and sensitivity analyses |
| Discussion | | |
| Key results | 18 | Summarise key results with reference to study objectives  “The present results show that the proportion of ZIKV-positive women was significantly greater in precarious women overall, and mostly in undocumented foreign women. Women living beyond the Coastal areas, where vector control is not as developed as in urban areas, were also significantly more likely to be infected by ZIKV.” |
| Limitations | 19 | Discuss limitations of the study, taking into account sources of potential bias or imprecision. Discuss both direction and magnitude of any potential bias  “The present study was hospital-based and the exact health insurance status was not always recorded for different reasons: lack of time in a busy obstetrical ward, communication problems because of the very large number of women who do not speak French. Living area used for the analysis did not allow to precisely study confusion between individual and collective socio-economic determinants. Despite these limitations, the present results were not a quest for any significant p value but were a clearly defined a priori hypothesis that was tested with the available data from the 2 biggest maternities in French Guiana, which capture most deliveries” |
| Interpretation | 20 | Give a cautious overall interpretation of results considering objectives, limitations, multiplicity of analyses, results from similar studies, and other relevant evidence  “The present results emphasize that population approaches for a range of selected problems may be more pertinent than an array of vertical social programs in different populations. Relationship between social aspects and seropositivity to ZIKV, to DENV (p<10−12), and to CHIKV (p<10−15) [12] [13] suggests a complex interplay between individual factors and ecological/environmental factors. Further studies should aim precisely define the potential multilevel causal paths underpinning this statistical association.  In conclusion, during the ZIKV epidemic in French Guiana, precarious pregnant women and women living in the western were significantly more affected by ZIKV than non-precarious women and women living in Central coastal areas.  ” |
| Generalisability | 21 | Discuss the generalisability (external validity) of the study results  The present results emphasize that population approaches for a range of selected problems may be more pertinent than an array of vertical social programs in different populations. Relationship between social aspects and seropositivity to ZIKV, to DENV (p<10−12), and to CHIKV (p<10−15) [12] [13] suggests a complex interplay between individual factors and ecological/environmental factors. Further studies should aim precisely define the potential multilevel causal paths underpinning this statistical association. |
| Other information | | |
| Funding | 22 | Give the source of funding and the role of the funders for the present study and, if applicable, for the original study on which the present article is based |

*Give information separately for cases and controls in case-control studies and, if applicable, for exposed and unexposed groups in cohort and cross-sectional studies.

**Note:** An Explanation and Elaboration article discusses each checklist item and gives methodological background and published examples of transparent reporting. The STROBE checklist is best used in conjunction with this article (freely available on the Web sites of PLoS Medicine at http://www.plosmedicine.org/, Annals of Internal Medicine at http://www.annals.org/, and Epidemiology at http://www.epidem.com/). Information on the STROBE Initiative is available at www.strobe-statement.org.
