# Supplementary material for: ZIKA Virus infection in pregnant women in French Guiana: More precarious-more at risk
Source: PLoS Negl Trop Dis. 2020 Mar 24;14(3):e0008193. doi: 10.1371/journal.pntd.0008193 (PMC7122809; doi:10.1371/journal.pntd.0008193)
Supplement: Attachment — Submitted filename: Dear Editors ZIKA PLoSNTDS (1).docx [file pntd.0008193.s002.docx]

***Dear Editors,***

***We are very grateful for the reviewer comments which have been helpful to look at our manuscript with fresh eyes. We have tried to follow their recommendations. You will find our responses inserted following the comments and suggestions.***

***We hope you will find the revised version has sufficiently improved.***

Overall the reviews were favorable, however, we cannot make any decision about publication until we have seen the revised manuscript and your response to the reviewers' comments. Your revised manuscript is also likely to be sent to reviewers for further evaluation.
 


Reviewer #1: The objectives of the study are clear. The study design and the population have to be more precisely detailed. The sample size is large. Analysis is correct but lack go adjustment. No concern about ethical or regulatory requirements.

Reviewer #2: The objectives of this study are clearly stated in the the hypothesis "that in pregnant women Zika Virus (ZIKV) was more frequent in precarious women than in non-precarious women as reflected by their health insurance status". The analytic approach supports the aims of the hypothesis.

However, the stated study design of "a retrospective case-control" is incorrect in reference to the study sample and analytic approach. The sample of of "all deliveries or medical abortions between January 2016 and December 2016" and the use of prevalence ratios suggest that a "cross-sectional study design" better describes this work. The sample was taken from a singular point in time. The authors then estimate the association between disease prevalence (ZIKV serology) and other prevalent health factors (precariousness via health insurance status) at that point in time. All of which is suggestion of a cross sectional study, not a case control.

***=>Yes we agree it is in fact a cross sectional design thanks for correcting us. We have modified.***

Reviewer #3: How can we be sure that woment with regular health insurance are not precarious?

***=>indeed this is true. Some French women may be precarious however, given the fact that CMU and AME are linked to a minimum income threshold, the type of health insurance is a good proxy for precariousness.***
The reason why neutralization for IgG could not be implemented should be explained.

***=>in this epidemic context resources were overstretched and seroneutralization would have been expensive and time-consuming we added an explanation in the methods. “Neutralization for IgG could not be implemented because it was time and resource-consuming in an epidemic context where human resources were overstretched, therefore we excluded probable cases with negative IgM and positive IgG.”***

Why is there no mention of an ethical committee  for this study?

***=>This is data from a national reference center which is an important official provider of epidemiological data. In the methods we have specified that the analysis of retrospective data did not require an ethical committee as it falls under retrospective analysis of anonymized data.***

--------------------


Reviewer #1: Analysis match the analysis plan but the results have to be completed to be comprehensive.

Reviewer #2: Overall, the analysis is clearly presented and the paper proceeds in a logical manner. However, the measure of association in the body of the paper, adjusted prevalence ratio (APR) does not match the measure in abstract (adjusted odds ratio, aOR). Please change the measure and language in the abstract (AOR, "associated with a higher risk of ZIKV") to match the body of the text (APR, "associated with a significant increase of ZIKV prevalence").
***=>thank you for spotting this remnant of an earlier version of the analysis we corrected.***
Reviewer #3: We need to understand who are the 837/6654 women who had positive IgG and negative IgM serology without immunocapture. It could represent a biais for the final analysis.

***=>Here under the notconfirmed=1 are these women.*** ***Overall 16.5% of precarious women had positive IgG and negative IgM serology without immunocapture whereas 9.82% of women with normal health insurance had positive IgG and negative IgM serology without immunocapture, P<0.001.***

The 2,621 women (39%) with unknown health insurance lived principally in Kourou
municipality (20%). This is a high rate, the risk that a high number of these women are precarious should be discussed.

***=>For kourou this is because we could not get the insurance variables from the hospital medical information department. We have specified.***
For abortion, please specify if there are spontaneous or not.

***=>in fact only spontaneous abortions or pregnancy termination for medical reasons were considered (voluntary abortions were not considered). We specified in the methods section***
It is stated that among precarious, irregular immigrants seemed more exposed 97/300 (32.3%) than French nationals 361/ 1310 (27.6%) (p=0.10). It need to be discussed further.

***=>ok undocumented women are more likely to be living in shantytowns with plenty of vector breeding sites, no municipal services or vector control whereas precarious women with residence permit may have slightly better living conditions. we have expanded in the discussion.***

--------------------


Reviewer #1: The conclusions are supported by the data. Limitations are clearly described but could be discussed further.

***=> we have expanded the limitations section for transparency “Although, the type of health insurance is based on residence permit and on having an income below a certain threshold, it is conceivable that health insurance as a proxy for social precariousness is not perfect and that some women in the regular health insurance group may have been precarious. The limited number of adjustment variables, notably the living area used for the analysis did not allow to precisely study confounding between individual and collective socio-economic determinants.”***

Reviewer #2: The conclusions are robust and link well to public health relevance in the context of the ZIKV epidemic in French Guiana.

The second to last paragraph could benefit from some clarifications:

- What specific vertical social programs are less beneficial than which population health approaches, in the context of preventing/addressing infectious disease in French Guiana?
**=>thank you it is indeed a bit obscure we have added the following sentence to try to clarify what we mean. *“Indeed, many community approaches are presently thematic (HIV, addictions…) and community worker often only focus on a single theme thus missing opportunities to improve outcomes for other important health problems.”***
- For the interplay between the individual factors of precariousness/poverty and the ecological/environmental factors resulting in the outcome of ZIKV as well as DENV and CHIKV:
~What are potential hypotheses for the possible associations or pathways resulting infection?

***=>we have tried to expand a bit. “At the individual level, underprivileged populations often have lower levels of health literacy; often live in shacks surrounded by* *mosquito-breeding grounds and little protection from vectors. At the ecological level these individual situations add up, and in addition these populations generally gather in informal habitats in areas that are more likely to have high vector densities, and to be less prioritized by municipal and regional services.”***

~How could these be verified and by what experimental designs?
~How could these future studies be used to inform public health in a manner that would mitigate or prevent the next infectious outbreak, especially among a vulnerable/precarious/impoverished population?

***=>thank you for the advice we have expanded a bit on the specifics” A better mapping of vector density in priority areas, specific knowledge attitudes and behavior studies in underprivileged populations, a survey of the geographic distribution of vector-control interventions, notably relative to underprivileged areas. Such data would allow devising interventions aiming at improving health literacy and empowering populations relative to the prevention of vector-borne diseases, and improving the reach of vector-control by specifically targeting such areas.”***

Reviewer #3: The epidemiology and the repartition of ZIKV infection in FG should be discussed. We need to know where most of the cases occured in this territory.
***=> the pregnant woman database is the most comprehensive data collection during the epidemic. This shows that western French Guiana was most affected this was mapped in “Flamand C, Fritzell C, Matheus S, Dueymes M, Carles G, Favre A, Enfissi A, Adde A, Demar M, Kazanji M, Cauchemez S, Rousset D. The proportion of asymptomatic infections and spectrum of disease among pregnant women infected by Zika virus: systematic monitoring in French Guiana, 2016. Eurosurveillance. 2017;22(44). doi:10.2807/1560-7917.ES.2017.22.44.17-00102.” We have specified this.***

--------------------

<b>Editorial and Data Presentation Modifications?</b></br><br/>
Use  this section for editorial suggestions as well as relatively minor modifications of existing data that would enhance clarity. If the only modifications needed are minor and/or editorial, you may wish to recommend “Minor Revision” or “Accept”.

Reviewer #1: Add a table with description of women by study categories.

***=>ok we have added a descriptive table***

Reviewer #2: - Change the study design from case-control to cross-sectional.
- Change the measure of association in the abstract from AOR to APR, remove reference to the risk of ZIKV and add a reference to the prevalence of ZIKV.

***=>ok this is indeed more precise we replaced throughout the text***
- Clarify some of the language and phrasing in the discussion section.

Please refer to the suggested minor modifications in the above sections for more details.

Reviewer #3: There is no author listed with affiliation 4 Regional epidemiology unit of French Public Health Agency, Cayenne, French Guiana.
***=>we corrected thanks for noticing***
--------------------


Reviewer #1: General comments
This study addresses an important topic in an area where data are limited: Zika virus infection in pregnant women in a French oversea territory located in the Amazon area and its relation to social insecurity. The proportion of women affected by Zika infection is very high and women in precarious situations seem more often concerned.
Although the study is monocentric, the number of pregnant women included is large.
In view of the existence in France of health insurance coverage for the poor and the undocumented (Complémentaire Santé Solidaire -CSS- and Aide Médicale d’Etat – AME-), the status relating to health insurance is a good proxy for measuring precariousness. However, it could be useful to discuss the lack of more precise data on the social situation. In particular, how were people without health insurance coverage treated?
As the rights to CSS and AME run 12 months regardless of changes in administrative status, the social situation of people may be different than that announced (undocumented migrants, precarious and non-precarious French resident )

***=>Women without health insurance can receive care (including ZIKV screening) at the mother and child clinics or they could receive free care after receiving a “PASS” voucher which allows covering the expenses. Thank you for asking it is not obvious for non-French readers. We have specified this in the methods***

Specific comments for revision
Title:
Zika Virus should be written in full in the title

***=>ok we modified***
Abstract:
Avoid repetitions “Most pregnant women were precarious 2275/3439. Most women were precarious but…”

***=>ok we have rephrased***
Please specify the number of women belonging to the 3 analysis categories (undocumented, precarious, non-precarious)

***=>ok we have added these numbers in the abstract***
Avoid “as elsewhere,” in the conclusion.

***=>ok, we have removed it***
In addition to the inequalities of vector control by neighborhoods, it would be interesting to question the differences in personal behavior in the application of these same measures

Manuscript:
Authors’ affiliations: an affiliation does not correspond to any author (4 Regional epidemiology unit of French Public Health Agency, Cayenne, French Guiana)

***=>thank you for noticing this error we corrected***
Detail abbreviations: GDP

***=>ok***

Between the summary which speaks of a monocentric study and the results which speak of 3 sites, it is not clear who was included in this study
***=>thanks for noticing we rephrased***

Corrected “assurance”.
There are inaccuracies to correct on French health insurance coverage
The Universal health coverage (=CMU) (basic health insurance coverage implemented in 1999) no longer exists since 2016, it was merged with national Health Insurance (Assurance maladie) during the Universal Health Protection reform (Protection universelle maladie -Puma-). In addition, the CMU does not have a 3-month residency obligation in France, unlike the AME.
The complementary universal health coverage (=CMUC) changed its name on 1/11/2019 when it was merged with Aid to complementary health (=ACS) and is now called complementary health solidarity (Complémentaire Santé Solidaire = CSS)
In addition, the CMU does not have a 3-month residency obligation in France, unlike the AME
Please specify for CSS and AME the resource criteria which explain why they can be used as a proxy for their social status
Prefer the term national Health Insurance (Assurance maladie) to that of social security (Sécurité Sociale) for others

**=> thank you for this observation.**

**After checking with our social worker indeed the switch from CMU to PUMa (it takes time to shake off the verbal habit to use the CMU word…) also occurred in 2016 in French Guiana so we modified. The CSS however, is much more recent and did not exist yet at the time of the epidemic. As suggested we added the financial thresholds to be more precise.**

The paragraph on the biological diagnosis must be reviewed and clarified

**=>Thank you is was very badly written. We tried to make it clearer.**
Avoid “ extraordinary context”

***=>replaced by emergency***

The methods of collecting data must be more precisely described. Likewise, it is not clear which motherhood participated and why.
A flow chart specifying the site for monitoring women and their health coverage would be welcome

***=>All women were followed in one of the 3 hospitals’ maternity wards (flow chart) we have rephrased to clarify.***
If prevalence ratio from Poisson regression were used, why in the abstract we found Odds Ratio?

***=> it is a remnant from a previous logistic regression analysis. we corrected.***

The procedures for diagnosing Zika infection should be described much more precisely:% of women diagnosed with PCR,% of women diagnosed with serology and in what term, with what result of the previous serology, etc. A detailed table would be welcome. These results will ideally be presented according to the 3 study categories

***=> ok we added serologies and pcr in a new table 1***

“and no insurance: APR = 1.59”?  You did not specify before having included people without health insurance. What are they? Profile?

***=>They were mentioned in the second sentence after the title health insurance status. We have added a table to describe.***

Table 1: detail the abbreviations at the bottom of the table. Specify the method of analysis in the title.

***=>ok we have added the table foot notes and modified the title***

Why not have adjusted model on age? Other adjustment variables, if available, would be welcome. The model as it is is difficult to interpret.

***=>we tried to be parsimonious so we had removed age. We added age categories in the multivariate model. This did not change anything .***

Discussion:
Can you discuss further why vector control measures are less implemented in certain regions and the policy of the regional health agency on this point. In particular, a qualitative insight into the real situation in the districts concerned would be useful for understanding the results.
The discussion could be enriched with international references having investigated the relationships between arboviruses and social situation

***=>we have expanded the discussion a bit just before the conclusion and added some additional references.***

Reviewer #2: This is an interesting analysis acceptable for publication following minor revisions. One minor thematic point relates on the authors comments on "The unusual distribution of mircocephaly in Brazil, suggested a possible link between poverty and the risk of ZIKV related microcephaly..." The association between poverty and increased risk of infectious disease should not be considered unusual. Especially given the authors prior notes concerning the lack of sanitation and presence of disease carring insects in poor neighborhoods in French Guiana.

***=>we agree we removed unusual***

Reviewer #3: This an interesting study that highlights the fact that precarious people are more at risk of Zika infection in French Guiana. We need to better understand the methodology and the recommandation for public Health.

***=>we have developped methodology and recommendations for public health.***
