# Supplementary material for: ZIKA Virus infection in pregnant women in French Guiana: More precarious-more at risk
Source: PLoS Negl Trop Dis. 2020 Mar 24;14(3):e0008193. doi: 10.1371/journal.pntd.0008193 (PMC7122809; doi:10.1371/journal.pntd.0008193)
Supplement: Attachment — Submitted filename: Dear Editor.docx [file pntd.0008193.s004.docx]

Dear Editor,

Thank you for your suggestions which we have accepted.

Regarding your point on this unclear sentence “We will subsequently refer to these two communities of communes” we rephrased, expanded a bit, and reordered from west to east for clarity. Indeed it wasn’t clear we hope it is better now.

We hope you will find the changes satisfactory

Best wishes

The authors
